# Predictors of Maternal Near Miss in Public Hospitals of West Shoa Zone, Central Ethiopia: A Case-Control Study

**DOI:** 10.3389/fmed.2022.868992

**Published:** 2022-04-29

**Authors:** Kababa Temesgen Danusa, Bikila Tefera Debelo, Negash Wakgari, Benyam Seifu, Ketema Kenasa, Gurmesa Daba, Fikadu Wondimu, Keneni Berhanu

**Affiliations:** Department of Midwifery, College of Medicine and Health Sciences, Ambo University, Ambo, Ethiopia

**Keywords:** maternal, near miss, predictors, West Shoa Zone, Ethiopia

## Abstract

**Background:**

Maternal mortality reduction remains a priority to ensure healthy lives and promote wellbeing for mothers and newborns in the new sustainable development goals agenda. There is no evidence-based study done regarding maternal complications and near-miss in the study area.

**Objectives:**

This study assessed the predictors of maternal near-miss in public hospitals of West Shoa Zone, Central Ethiopia, 2020.

**Methods:**

An unmatched case-control study was conducted among 664 (166 cases and 498 controls) women who gave birth in public institutions in the West Shewa zone. Structured questionnaires and checklists were used to collect the data. Bivariate, multivariable logistic regression, and adjusted odds ratios were used to describe the strength and directions of association.

**Results:**

The odds of maternal near-miss were higher among mothers with increased maternal age [Adjusted odds ratio (AOR) = 1.065, 95%CI: (1.015–1.117)], who could not read and write (AOR = 3.06, 95%CI: 1.314–7.135), had primary (AOR = 3.49, 95%CI: 1.518–8.044), and secondary (AOR = 3.213, 95%CI: 1.418–7.282), had no antenatal care (ANC) follow-up (AOR = 2.25, 95%CI: 1.100–4.607), mothers who had a first delay of more than 6 h [AOR = 2.38, 95%CI: (1.517–3.735)] and the distance from health facility of > 60 min [AOR = 4.021, 95%CI: (1.817–8.896)].

**Conclusion:**

In this study, delay in decision making and reaching the health facility, lower educational status, not having ANC follow-up, and increased maternal age were significantly associated with maternal near misses. Therefore, the Ethiopian federal ministry of health and other stakeholders should work on increasing ANC coverage, awareness creation, and strong means of transportation to tackle the complications of a maternal near miss.

## Background

Maternal and neonatal mortality and morbidity reduction remain a priority to ensure healthy lives and promote wellbeing for mothers and newborns in the sustainable development goals agenda (1). Maternal near-miss is mothers who almost passed away but survived from a complication that occurred during pregnancy, intrapartum, or within 42 days of termination of pregnancy (2). Maternal near-miss events vary globally from 0.14 to 0.75% in high-income countries, 1.5–7.7% in middle-income countries, and 2.21–12% in sub-Saharan countries (3–5).

Globally, pregnancy-induced hypertension, bleeding, infection, anemia, and labor dystocia were documented as a direct cause of maternal near-miss (6–8). Maternal near-miss may not necessarily be a result of direct causes. According to different studies, factors like advanced maternal age, lower socioeconomic status, less or no antenatal care (ANC), preexisting medical conditions, and three delays could aggravate women’s morbidity experiences (7, 9–13). In Ethiopia, harmful traditional views and practices, lack of infrastructure, lack of transportation, insufficient care at the facility, weak referral system, scarcities of supplies, delay in recognizing danger signs, and deciding to seek care, and high parity level may lead to mortality or serious morbidity experiences (14, 15). Besides, a study done in southwest Ethiopia shows that there were 5,530 live births, 210 maternal near-misses, 17 maternal deaths, and maternal near-miss is 24.85% (9).

Ethiopia is amongst the low-income sub-Saharan African countries with the highest maternal mortality rate which is 412 per 100,000 live births, and for each maternal death, up to 15% of the women develop disability from pregnancy-related complications. Though in most of the health institutions of Ethiopia maternal mortality ratios are high, the absolute number per health center is rare which leads to the low power of the studies to consider the potential predictors (16). Therefore, maternal near-miss could serve as an indicator for maternal death to assess the quality of obstetric care in specific health institutions (2, 17). Thus, it is important to study maternal near-misses or severe acute maternal morbidity as a complement to evaluating and improving the quality of obstetric care. Hence, this study assessed predictors of maternal near-miss in public hospitals of West Shoa Zone, Central Ethiopia.

## Materials and Methods

### Study Setting, Design, and Population

An unmatched case-control study was employed amongst women of childbearing age (15–49) in West Shoa Zone public Hospitals, Ethiopia from June to October 2019. The West Shoa Zone is located at a 126 km distance from Addis Ababa, the capital city of Ethiopia to the west, and its capital is Ambo town. There are around 495,753 reproductive-age women and about 100,283 mothers who gave birth per year in this zone. In this zone, there are eight public hospitals, 92 health centers, and 528 health posts.

### Sample Size Determinations

The sample size was estimated using Epi info V.3.5.1 software depending on the following parameters: 95% confidence level, power of 80%, case to control the ratio of 1:3, percentage controls exposed of l4.11%, and percent of cases with exposure of 10.78% (9). With 10% non-response, the final sample size becomes 664. Hence, 166 cases and 498 controls were considered for the study.

### Sampling Procedure

Cases were identified by using modified World Health Organization (WHO) criteria to classify maternal near-miss who were admitted for childbirth or management of pregnancy-related problems or within 42 days of delivery to all public hospitals in the study settings. A mother with any of the conditions that are stated in the modified WHO criteria of maternal near-miss was selected as a case in this study (Table 1) (2, 18). To capture more cases, clinical criteria for severe maternal complications such as severe preeclampsia, Eclampsia, severe systemic infection, and ruptured uterus have been added to the WHO criteria as these show fatal maternal complications and were not addressed in the WHO criteria. Controls were those mothers admitted to similar Hospitals in the study setting but without complications during pregnancy, childbirth, or within 42 days of delivery and minor problems that are not classified under maternal near-miss cases by the modified WHO criteria. Based on the number of women admitted with the maternal near-miss and without maternal near-miss 1 year before the study, both cases and controls were proportionately assigned to each hospital. Mothers who were admitted to a similar hospital where the cases transpired and were delivered without complication or those who were not showing any severe pregnancy-related complications were enrolled as a control. Three controls per case were selected to increase the power of the study to detect any difference in the determinants between cases and controls. A lottery method was used to select the controls among eligible women.

**TABLE 1:** WHO maternal near-miss criteria adapted to the local context of low-resource-setting [reproduced from Nelissen et al. (18)].

WHO near-miss criteria	Haydom near-miss criteria
**Clinical criteria**	
Acute cyanosis	Acute cyanosis
Gasping	Gasping
Respiratory rate > 40 or <6/min	Respiratory rate >40 or <6/min
Shock	Shock^a^
Oliguria non-responsive to fluids or diuretics	Oliguria non-responsive to fluids or diuretics^b^
Failure to form clots	Failure to form clots^c^
Loss of consciousness lasting >12 h	Loss of consciousness lasting > 12 h^d^
Cardiac arrest	Cardiac arrest^e^
Stroke	Stroke^f^
Uncontrollable fit/total paralysis	Uncontrollable fit/total paralysis^g^
Jaundice in the presence of pre-eclampsia	Jaundice in the presence of pre-eclampsia^h^
**Laboratory-based criteria**	
Oxygen saturation <90% for ≥60 min	Oxygen saturation <90% for ≥60 min
PaO2/FiO2 < 200 mmHg	
Creatinine ≥ 300 μmol/l or ≥ 3.5 mg/dL	
Bilirubin > 100 μmol/l or > 6.0 mg/dL	
pH < 7.1	
Lactate > 5 mEq/mL	
Acute thrombocytopenia (<50,000 platelets/ml)	Acute thrombocytopenia (<50,000 platelets/ml)
Loss of consciousness and ketoacids in urine	
**Management-based criteria**	
	Admission to an intensive care unit
Use of continuous vasoactive drugs	
Hysterectomy following infection or hemorrhage	Hysterectomy following infection or hemorrhage
Transfusion of ≥ 5 units of blood	Transfusion of ≥ 1 unit of blood
Intubation and ventilation for ≥ 60 min are not related to anesthesia	Intubation and ventilation for ≥ 60 min are not related to anesthesia
Dialysis for acute renal failure	
Cardiopulmonary resuscitation	Cardiopulmonary resuscitation
**Severe maternal complications**	
	Eclampsia^i^
	Sepsis or severe systemic infection^j^
	Uterine rupture^k^

*^a^Shock is defined as persistent severe hypotension, defined as a systolic blood pressure < 90 mmHg for 60 min with a pulse rate of ≥ 120/min despite aggressive fluid replacement (> 2 L).*

*^b^Oliguria is defined as a urinary output of < 30 ml/h for 4 h or < 400 ml/24 h.*

*^c^Failure to form clots is defined as the absence of clotting from the IV site after 7–10 min.*

*^d^Unconsciousness/coma lasting > 12 h is defined as a profound alteration of mental state that involves a complete or near-complete lack of responsiveness to external stimuli or Glasgow Coma Scale < 10.*

*^e^Cardiac arrest is defined as loss of consciousness and absence of pulse or heartbeat.*

*^f^Stroke is defined as a neurological deficit of cerebrovascular cause that persists ≥ 24 h, or is interrupted by death within 24 h.*

*^g^Uncontrollable fit is a condition in which the brain is in a state of continuous seizure.*

*^h^Pre-eclampsia: the presence of hypertension associated with proteinuria. Hypertension is defined as blood pressure ≥ 140 mmHg (systolic) or ≥ 90 mmHg (diastolic). Proteinuria is defined as the excretion of ≥ 300 mg protein/24 h or 300 mg protein/liter urine or ≥ 1 + on a dipstick.*

*^i^Eclampsia is defined as the presence of hypertension associated with proteinuria and fits. Hypertension is defined as blood pressure ≥ 140 mmHg (systolic) or ≥ 90 mmHg (diastolic). Proteinuria is defined as the excretion of ≥ 300 mg protein/24 hr or 300 mg protein/liter urine or ≥ 1 + on a dipstick.*

*^j^Sepsis is defined as a clinical sign of infection and 3 of the following: temp > 38°C or < 36°C, respiration rate > 20/min, pulse rate > 90/min, WBC > 12.*

*^k^Uterine rupture is defined as the complete rupture of a uterus (including peritoneum) with (partial) extrusion of the fetus during labor” (2, 19).*

### Operational Definitions

**Maternal near-miss:-** A woman who almost passed away but survived a complication that happened during pregnancy, childbirth, or within 42 days of delivery (18).

**Delay one:** Mothers delay in recognizing danger signs and deciding to pursue medical attention.

**Delay two:** Mothers delay in reaching an appropriate health facility to get health care services.

### Data Collection Tools and Quality Control

After the near-miss cases and controls have been identified, all the mothers who escaped maternal death, as well as those who deliver without complications, were interviewed (Exit-interview) using a pre-tested and structured questionnaire. Similarly, data extraction was also employed by using checklists from maternal hospital medical records for certain variables. Five data collectors and two supervisors were selected for the data collection. Data collection tools contain socioeconomic and demographic characteristics of the respondents, reproductive health services, obstetric history, and preexisting medical conditions.

Data quality was assured throughout the collection, coding, entry, and analysis of the data. A 2-day training was provided for the data collectors and supervisors before actual data collection. The collected data were revised and checked for uniformity, clearness, wholeness, and accuracy throughout the data collection process by data collectors and supervisors. Furthermore, a pretest was conducted among 5% of the sample and the result was used to amend the tool before the actual data collection and was not used in the final analysis.

### Data Analysis

Both bivariate and multivariable logistic regressions were fitted to assess the significant factors. The case and control groups were compared based on socio-economic and demographic characteristics, obstetric history, access to reproductive health services, and preexisting medical conditions. Variables with a *p*-value of less than 0.2 in the bivariate analysis were included in the final multivariable logistic regression analysis with an exception of independent variables showing multicollinearity (Pearson coefficient greater than 0.7 or less than -0.7). Consequently, “family size” correlated with “total number of pregnancies” (Pearson correlation coefficient 0.722) and distance from health facility and delay two (Pearson correlation coefficient 0.7). Additionally, “occupation of the mother” and “monthly income” were removed from the final analysis after showing the significant difference and implausible odds in the analysis. Hosmer and Lemeshow’s goodness of fit test was used to check model fitness before running the final model. Finally, screened variables were fitted to the multivariable logistic regression model through a backward stepwise method to reduce the effects of cofounders and to identify the independent effects of each variable on the outcome variable. Adjusted odds ratios with a 95% confidence interval and *p* < 0.05 were reported to declare the predictors of the maternal near miss.

## Results

### Socio-Demographic Characteristics

In the current study, a total of 664 mothers, 166 cases, and 498 controls, were interviewed with a full response rate with no refusal. Regarding age, more than one-third, 180 (36.1%) of the controls were aged between 20 and 24 years whereas 47 (28,3%) of the cases were 35–39 years of age. More than half, 293 (58.8%) of the control group were urban residents whereas in the majority of the cases 94 56.6%) were rural residents. More than one quarter, that is, 43 (25.9%) of the cases and 154 (30.9%) of the controls had secondary education (Table 2).

**TABLE 2:** Distribution of socio-demographic characteristics over maternal near-miss in West Shoa Zone public hospitals, Central Ethiopia, 2020.

Characteristics	Controls (%)	Cases (%)
**Maternal age**		
< 19 years	40 (8)	5 (3)
20–24 years	180 (36.1)	34 (20.5)
25–29 years	141 (28.3)	38 (22.9)
30–34 years	66 (13.3)	37 (22.3)
35–39 years	48 (9.6)	47 (28.3)
40 years and above	23 (4.6)	5 (3)
**Marital status**		
Married	473 (95)	165 (99.5)
Others*	25 (5)	1 (0.5)
**Maternal education**		
Cannot read and write	132 (26.5)	75 (45.2)
Primary education	122 (24.5)	40 (24.1)
Secondary education	154 (30.9)	43 (25.9)
College and above	90 (18.1)	8 (4.8)
**Place of residence**		
Urban	293 (58.8)	72 (43.4)
Rural	205 (41.2)	94 (56.6)
**Monthly income (ETB)**		
<500	124 (24.9)	52 (31.3)
500–1,000	126 (25.3)	24 (14.5)
1,000–2,000	95 (19.1)	45 (27.1)
>2,000	153 (30.7)	45 (27.1)
**Maternal occupation**		
Housewife	227 (45.6)	89 (53.6)
Government employee	77 (15.5)	7 (4.2)
Self-employed	90 (18.1)	24 (14.5)
Student	32 (6.4)	12 (7.2)
Farmer	72 (14.5)	34 (20.5)
**Husband’s occupation**		
Government Employee	140 (28.1)	38 (22.9)
Self-employed	153 (30.7)	27 (16.3)
Student	7 (1.4)	5 (3)
Farmer	173 (34.7)	95 (57.2)
**Distance from the health facility (walking time)**		
≤30 min	267 (53.6)	51 (30.7)
30–60 min	117 (23.5)	42 (25.3)
≥60 min	114 (22.9)	73 (44.)

### Obstetrics Characteristics Over a Maternal Near-Miss

**FIGURE 1:**
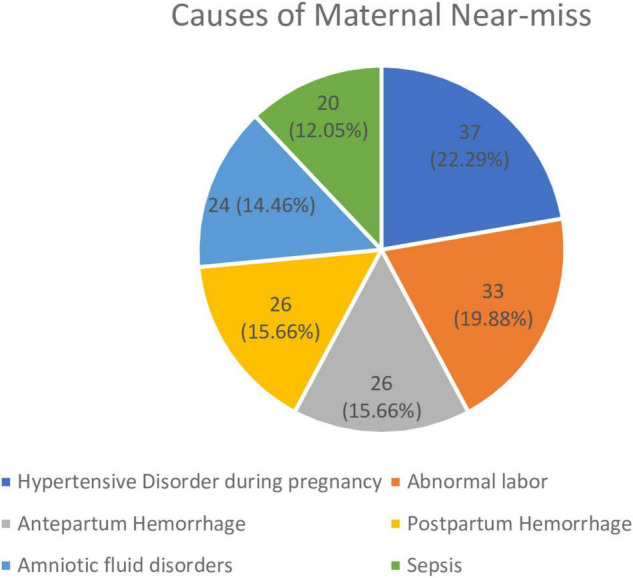
Causes of maternal near-miss during pregnancy, childbirth, and postpartum period in West Shewa Public Health Institutions, Ethiopia, 2020 (*N* = 166).

Mostly, 160 (96.4%) of near-miss cases happened before hospital admission. About 95% of the control and 87.3% of the cases group have had ANC follow-up (Table 3). Regarding the cause of maternal near-miss, hypertensive disorders during pregnancy were the major cause 37 (22.29%) followed by abnormal labor 33 (19.88%), antepartum hemorrhage 26 (15.66%), postpartum hemorrhage 26 (15.66%), amniotic fluid disorders 24 (14.46%), and sepsis 20 (12.05%) (Figure 1).

**TABLE 3:** Distribution of obstetric characteristics over maternal near-miss in West Shoa zone public hospitals, Central Ethiopia, 2020.

Characteristics	Control (%)	Cases (%)
ANC follow up*		
Yes	472 (94.7)	145 (87.3)
No	26 (5.3)	21 (12.7)
**Number of ANC visit**		
One	47 (10)	4 (2.8)
Two	32 (6.8)	38 (26.2)
Three	182 (38.6)	61 (42.1)
Four and above	211 (44.7)	42 (29)
**Number of alive children**		
None	43 (8.6)	21 (12.7)
One	204 (41)	36 (21.7)
Two to four	177 (35.5)	67 (40.4)
Five and above	74 (14.9)	42 (8.4)
**Number of total pregnancies**		
One	186 (37.3)	34 (20.5)
Two to four	216 (43.4)	67 (40.4)
Five and above	96 (19.3)	65 (39.2)
**History of stillbirth**		
Yes	54 (10.8)	33 (19.9)
No	444 (89.2)	133 (80.1)
**Bad obstetric history**		
Yes	67 (13.5)	20 (22)
No	431 (86.5)	146 (88)
**History of abortion**		
Yes	73 (14.7)	32 (19.3)
No	425 (83.3)	134 (80.7)
**Contraceptive history**		
Yes	367 (73.7)	122 (73.5)
No	131 (26.3)	44 (26.5)
**History of chronic disease**		
Yes	68 (13.7)	22 (13.3)
No	430 (86.3)	144 (86.7)

*A mother was classified as having ANC follow-up if she had visited a health facility at least once for pregnancy-related follow-up.

### Predictors of Maternal Near-Miss

Mothers’ age, education, residence, ANC follow-up, previous total pregnancy, stillbirth history, distance from a health facility, delay at home in making decisions (first delay), and husbands’ occupation were predictors of a maternal near-miss in bivariate analysis and taken to the multivariable logistic regression. However, mothers’ age, education, first, and distance from health facility remained to be predictors of maternal near-miss in multivariable logistic regression.

The odds of maternal near-miss were about 3, 3.4, and 3.2 times higher among mothers who cannot read and write [Adjusted odds ratio (AOR) = 3.06, 95%CI: (1.314–7.135)], had primary [AOR = 3.49, 95%CI: (1.518–8.044)], and secondary education [AOR = 3.213, 95%CI: (1.418–7.282)] than those who had college and above education, respectively. Similarly, for a year increase in age of a woman, the odds of maternal near-miss increase by a factor of 1.065 [AOR = 1.065, 95%CI: (1.015–0.117)]. Moreover, the odds of maternal near-miss were more than two times higher among mothers who had not had ANC follow-up during the current pregnancy [AOR = 2.25, 95%CI: (1.100–4.607)] than those who had ANC follow-up. Further, the odds of maternal near-miss among mothers who had a first delay of more than 6 h at home in deciding to seek health care were more than twice higher than those who had a first delay of fewer than 6 h [AOR = 2.38, 95%CI (1.517–3.735)]. Finally, walking distance from a health facility of more than an hour is associated with about 4 times higher odds of maternal near-miss [AOR = 4.021, 95%CI (1.817–8.896)] (Table 4).

**TABLE 4:** Bivariate and multivariable analysis of maternal near-miss predictors in West Shoa zone public hospitals, Central Ethiopia, 2020.

Variables	Maternal Near-miss		Crude OR [95% CI]	Adjusted OR [95%CI]	*P*-value
	
	No	Yes			
**Age**			1.084 (1.054–1.114)	**1.065 (1.015–1.117)**	**0.010**
**Husband occupation**					
Government employee	140	38	1	1	1
Self employed	160	32	0.73 (0.43–1.24)	0.639 (363–1.124)	0.120
Farmer	173	95	2.02 (1.30–3.13)	0.932 (0.399–2.173)	0.870
**Mothers’ educational status**					
Cannot read and write	132	75	6.39 (2.94–13.90)	**3.062 (1.314–7.135)**	0.000
Primary education	122	40	3.69 (1.65–8.26)	**3.494 (1.518–8.044)**	0.003
Secondary education	154	43	3.14 (1.41–6.98)	**3.213 (1.418–7.282)**	0.005
College and above	90	8	1	1	1
**Place of residence**					
Urban	293	72	1	1	1
Rural	205	94	1.87 (1.31–2.66)	0.685 (0.398–1.180)	0.173
**Distance of health facility (walking time)**					
≤30 min	267	51	1	1	1
30–60 min	117	42	1.88 (1.18–2.99)	1.699 (0.845–3.417)	0.137
≥60 min	114	73	3.35 (2.20–5.50)	**4.021 (1.817–8.896)**	**0.001**
ANCvisits*					
Yes	472	145	1	1	
No	26	21	2.629 (1.437–4.812)	**2.251 (1.100–4.607)**	**0.026**
**Total number of pregnancies**					
One	186	34	1	1	1
Two to four	216	67	1.70 (1.08–2.68)	1.237 (0.708–2.162)	0.454
Five and more	96	65	3.70 (2.29–6.00)	1.414 (0.667–2.997)	0.366
**Still Birth**					
Yes	54	33	2.04 (1.27–3.28)	1.181 (0.679–2.053)	0.556
No	444	133	1	1	1
**Delay one**					
>6 h	88	52	2.13 (1.42–3.17)	**2.380 (1.517–3.735)**	**0.000**
<6 h	410	114	1	1	1

*A mother was classified as having ANC follow-up if she had visited a health facility at least once for pregnancy-related follow-up. Adjusted for: Mothers’ age, educational status, place of residence, ANC follow-up, previous total pregnancy, stillbirth history, distance from a health facility, delay at home in making decisions (first delay), and husbands’ occupation.

*Bold values are significantly associated at a p-value of <0.05.*

## Discussion

This study assessed determinants of a maternal near-miss in public hospitals of West Shoa Zone, Central Ethiopia. Hypertensive disorders during pregnancy including preeclampsia with severe features and eclampsia are among the common causes of maternal mortality in resource-poor settings like Ethiopia and therefore, should be incorporated into the maternal-near-miss classification by updating to the local context.

According to this study, for a year increase in age of a woman, the odds of maternal near-miss increase by a factor of 1.065. A multi-setting study done in Brazil that reported the risk of maternal near-miss increased by about a quarter in older women corroborates this finding (19). Advanced or increased maternal age is associated with adverse pregnancy outcomes which in turn could lead to severe maternal morbidity or maternal-near miss and mortality (20).

In this study, the odds of maternal near-miss among respondents who cannot read and write, and who had primary and secondary education are higher compared to those who had college and beyond education. This finding is in line with the studies done in Ethiopia (21, 22). In the same fashion, the evidence from the studies done in Brazil, Bolivia, and Morocco also reported that mothers with lower-level educational status are at increased risk for maternal near-miss (7, 23, 24). Studies indicated that general health outcome and literacy have a positive relationship which could be extended to this study explaining the higher odds of maternal near-miss among mothers with lower educational status (25).

Moreover, the odds of maternal near-miss were more than 2 times higher among mothers who had not had ANC follow-up during the current pregnancy. This is supported by a study done in Southwest Ethiopia (9), a systematic review and meta-analysis which concluded about three fourth of maternal near-miss events will be averted by at least one ANC visit (26). ANC is an opportunity for early detection and management of obstetric problems that could be life-threatening to the mother later during labor and delivery and the postpartum period. Causes of maternal near-miss identified in this study, such as hypertensive disorders during pregnancy, hemorrhage-related complications, and other obstetric-related problems can be decreased by ANC follow-up.

The longer the distance to reach a health facility to receive health care, the higher odds of maternal near-miss. This is consistent with the studies done in the Amhara region (27), Northern (21), and Western Ethiopia (22). This might be due to poor infrastructure and lack of transportation leading the mothers to delays in arriving at the medical care center with severe maternal complications (7, 9, 28).

Likewise, delay at home due to delay in decision-making to seek health care is another predictor of a maternal near miss. This is in line with the studies done in west Arsi (29) and the Gurage zone, Ethiopia (30), and Morocco (24). This might be because of a lack of awareness of pregnancy-related complications which could be a protective reason for a maternal near miss. If mothers delay recognizing danger signs during pregnancy, childbirth, postpartum, and fail to decide to seek health care quickly that might lead to morbidity and mortality (13, 31, 32). Obstetrics care is time-sensitive, poor recognition of clinical conditions is also indicated to delay in deciding to seek care further increasing the risk of getting timely care (33).

### Limitation

Due to the nature of data collection tools and time, recall bias could not be avoided.

## Conclusion

In this study, delay in decision making and reaching the health facility, lower educational status, not having ANC follow-up, and increased maternal age were significantly associated with maternal near misses. Therefore, the Ethiopian federal ministry of health and other stakeholders should work on increasing ANC coverage, awareness creation, and strong means of transportation to tackle the complications of a maternal near miss.

## Data Availability Statement

The raw data supporting the conclusions of this article will be made available by the authors, without undue reservation.

## Ethics Statement

The ethical clearance was obtained from an ethical review committee of the college of medicine and health sciences, Ambo University with the reference number AU/CMHS-RCS:226/2019. A letter of support was submitted to each hospital. Both written and verbal consent was obtained from each study subject before the data collection process proceeded and from legal representatives for minor participants. Written informed consent to participate in this study was provided by the participants’ legal guardian/next of kin.

## Author Contributions

KD, BD, and NW conceived, designed the study, analyzed the data, and wrote up the manuscript. BS, KK, GD, FW, and KB supervised, assisted in designing, and wrote up the manuscript. All authors read and approved the final manuscript.

## Conflict of Interest

The authors declare that the research was conducted in the absence of any commercial or financial relationships that could be construed as a potential conflict of interest.

## Publisher’s Note

All claims expressed in this article are solely those of the authors and do not necessarily represent those of their affiliated organizations, or those of the publisher, the editors and the reviewers. Any product that may be evaluated in this article, or claim that may be made by its manufacturer, is not guaranteed or endorsed by the publisher.
